# Guillain‐Barré syndrome in a man presenting with upper extremity monoplegia

**DOI:** 10.1002/ccr3.2613

**Published:** 2020-03-11

**Authors:** Jacqueline Marie Gowan, Abhinav Golla, Antonio Liu

**Affiliations:** ^1^ Mt Sinai Hospital Chicago IL USA; ^2^ Department of Neurology Adventist Health White Memorial Los Angeles CA USA

**Keywords:** autoimmune, Guillain‐Barré syndrome, monoplegia, neurology, paralysis

## Abstract

Outside academia, community and rural medical centers often rely on clinical signs and symptoms for early intervention, as means of gathering objective data may be unavailable, or the reliance of biological testing may take too long to return. Continued reporting of atypical presentations is therefore necessary for broadening clinical recognition of such cases.

## Graphical abstract

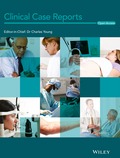

## 1 INTRODUCTION

Guillain‐Barré syndrome (GBS) is an antibody‐driven postinfective autoimmune disorder in which patients typically experience progressively symmetric ascending weakness. Here, we present an atypical case of GBS with initial asymmetric right arm weakness, descending progression, and respiratory compromise.

Guillain‐Barré syndrome (GBS) is a broad term used to describe a number of autoimmune inflammatory disorders targeting motor nerves, in which patients typically experience a predictable pattern of diminished strength symmetrically in the lower limbs with rapid ascending progression, coupled with hyporeflexia and ataxia over the course of 4‐28 days.^1^, ^2^

Although the pathogenesis of GBS is not understood in its entirety, the current theory favors the production of autoantibodies that target ganglioside macromolecules normally responsible for signal transduction, cell‐to‐cell communication, and growth.^2^ These ganglioside macromolecules are located according to their subset in different concentrations throughout the nervous system. For example, the Ga1NAc‐GD1a gangliosides are located in periaxonal membranes of motor nerves, whereas the GQ1b gangliosides are concentrated in large amounts in the myelin of the oculomotor, trochlear, and abducens nerves.^2^ Though the exact cause of GBS is not universally agreed upon, epidemiological studies suggest that as many as 75% of patients have preceding infectious symptoms^3^ (30% overall infected with Campylobacter jejuni),^3^ which favors a process related to molecular mimicry.^3^, ^4^ The immune system develops antibodies to antigens on the infectious agent that resemble gangliosides on nerve cells, resulting in a T‐cell/macrophage‐mediated autoimmune attack.^5^ The variation in regard to which specific ganglioside subtype these antibodies target is believed to play a role in both the pattern and location of weakness experienced by the patient.

Although the clinical presentation and predictable progression of classical GBS and its variants have been well‐documented, reports of atypical cases are sparse. This presents a problem, as a delay in initiation of treatment in patients with GBS increases the chance of prolonged ataxia and chronic weakness. The theory of molecular mimicry behind the pathogenesis of GBS may account for cases that follow bacterial, fungal, or parasitic infections; however, the hypothesis loses strength when GBS patients present after a viral illness, idiopathically, or in those who test negative for antiganglioside antibodies at all—yet still demonstrate evidence of axonal damage on electrophysiological studies.

For the academic world, it is important to continue to report cases of atypical GBS, as this may help to further build an understanding of the characteristics of the disease. For the medical world, reporting atypical GBS patients is important, as some settings may rely on a clinical diagnosis to initiate treatment, as objective means may not be readily available (such as electrophysiological studies in rural or community hospitals), or results from antibody studies may take over a week to return. Here, we present a case of rapid onset unilateral upper extremity weakness with caudal progression and eventual respiratory failure in a patient who tested positive for CSF antiganglioside IgG.

## 2 CASE REPORT

Our case follows a 55‐year‐old right‐handed man without significant medical history, who presented to our emergency department (ED) reporting consistent and worsening “heaviness” in his right arm for the past two days. At the time, the patient denied any other symptoms such as difficulty walking, pain, headache, or sensory changes. History revealed the patient had a “small cold” or upper respiratory illness 3 weeks prior, and he denied any diarrhea, recent vaccinations, travel history, or animal/farm contact. Vitals were significant for a blood pressure of 220/139. He was afebrile. Examination in the ED revealed a man with an appropriate mentation, alert, and orientated to person, place, and time. Uniform weakness was noted in the patient's right upper extremity (RUE), with a motor strength of 3/5. Reflexes were noted as 1+ throughout the RUE as well. Motor strength and reflexes in the left upper extremity (LUE) and in the bilateral lower extremities (BLEs) were 5/5 and 2+, respectively. No sensory impairment was noted. Cranial nerves were grossly intact throughout, without evidence of dysarthria, aphasia, or dysphagia. Patient's gait was unaffected, and he was noted to walk without assistance in the ED. Additional testing in the ED including urine toxicology, head CT, head MRI with diffusion‐weighted imaging, and cervical‐spine imaging which all showed no significant findings. Without an obvious diagnosis, the patient was admitted for observation.

The first two days of admission were uneventful, with the patient denying any change in weakness. Electrophysiological studies were unavailable at our institution, requiring us to rely on clinical symptoms and biological evidence from antibody studies for assessment and diagnosis. Lumbar puncture conducted on hospital day 2 rendered a CSF profile consisting of 1 white blood cell, no red blood cells, and 33 mg/dL of protein. Additionally, CSF was tested for antiganglioside antibodies as well as IgG/IgM antibodies against West Nile and Zika virus (although testing for the latter was denied by the Los Angeles County Health Department due to a lack of recent travel history/contact with travelers). Serum bioassay was negative for the botulism toxin.

By hospital day 3, the patient endorsed a decline in RUE motor strength in comparison with when he was examined in the ED (now 2/5 motor strength), as well as new LUE weakness and “heaviness” (motor strength of 4/5). Repeat MRI (on hospital day 3) remained negative for any pathology suggestive of transverse myelitis, or infiltrative disease. Bilaterally, strength in the lower extremities was spared until hospital day 4, when it was noted the patient was unable to walk unassisted. Additionally, on the fourth day the patient experienced complete flaccid paralysis of the RUE, continued decline in LUE strength (3/5 strength), and complete areflexia throughout the entirety of his extremities. As the patient's weakness worsened over the following days, he never developed significant ophthalmoplegia, nystagmus, ptosis, dysphagia, sensory loss, or change in mentation (remaining alert and orientated throughout). Of note, the patient was examined by the same neurologist each day in an attempt to keep the variation in subjective assignment of motor strength at a minimum.

Serum absorbance values for the anti‐GD1a antibody returned positive at 356nm, strongly favoring a diagnosis of GBS with atypical onset, and intravenous immunoglobulin (IVIG) was ordered. Unfortunately, the patient developed respiratory distress on hospital day 7, which required intubation and transfer to the intensive care unit. The patient received 5 days of IVIG therapy while in the ICU, with gradual improvement of his weakness noted on hospital day 14. The patient eventually required a tracheostomy and gastric tube placement while in the ICU, where he stayed for an additional 3 weeks until he was transferred to a skilled nursing facility. Eighteen months after his discharge, the patient returned to our ED for an unrelated issue, at which time he no longer had a tracheostomy or gastric tube, and was noted to ambulate with a cane.

## 3 DISCUSSION

Clinicians practicing in either community or rural hospitals often have to rely on signs and symptoms of GBS in order to make a diagnosis,^1^, ^6^ as objective means to gather data may be unavailable (such as electrophysiology—although helpful in the clinical setting, but not generally required for GBS diagnosis), or depend on third‐party laboratories—meaning a lengthy wait for results to return, potentially diminishing the chance of a full recovery. It is therefore imperative that physicians are able to recognize not only the classical variant of GBS, but also the other ways a GBS patient can present and initiate treatment, as delays are associated with long‐term ataxia and chronic weakness.^1^ To our knowledge, this is the first case report that specifically addresses a patient with suspected GBS who presented with asymmetric weakness in an upper extremity which progressed caudally to include all four limbs.

Rapidly progressive bilateral weakness is the main symptom a GBS patient will present with, typically ascending from the lower limbs to the upper extremities,^1^, ^2^, ^3^, ^4^, ^5^, ^6^, ^7^ although others may present with cranial nerve involvement, or with bulbar, or autonomic dysfunction at first. For diagnosis of GBS, progressive weakness and areflexia must be present.^7^ Nerve conduction studies (NCS) which are often used at higher‐level medical centers are certainly helpful in diagnosis, as they can better discriminate between axonal and demyelinating subtypes; however, clinicians should not rely completely on NCS, as differentiation between the subtypes has no effect on treatment—and may in fact delay treatment if relied on entirely for diagnosis. Additionally, the timing of NCS also plays a factor in the reliability of these tests as well—with the greatest response peaking 2 weeks after the initiation of weakness, and with the smallest/a normal response early on. NCS are helpful, however, in predicting prognosis in GBS patients, as those with features of demyelination and low compound muscle action potentials are often seen in those who will require mechanical ventilation.^7^

Once suspected on clinical grounds, differentiation of each of the GBS variants, be it classical, Miller‐Fisher, pharyngeal‐cervical‐brachial, or a mixture, is ultimately an academic pursuit. Each variant, however, presents differently in regard to the pattern of progression and therefore can cause confusion and delays in the initial diagnosis and management of GBS. Collection of objective data such as antibody LP testing can help confirm the diagnosis in uniquely presenting patients (such as our case), and publication of these data can help the scientific community to better understand this disease (as there are many aspects of the pathophysiology of GBS and its variants that are not completely understood). Here, we briefly highlight a few of the GBS subcategories, the different ways they have been known to present, along with their respective objective findings. To reiterate, treatment for each subtype is the same, and the goal of the clinician should be to identify those patients who may have GBS and initiate treatment as soon as possible.

## 4 CLASSICAL GBS (DEMYELINATING AND AXONAL)

Broadly speaking, classical GBS is divided into two primary categories, the demyelinating and the axonal. The demyelinating, referred to as acute inflammatory demyelinating polyneuropathy (AIDP), makes up 85%‐90% of all classical GBS cases in the Western Hemisphere and presents with symmetric lower limb involvement with rostral progression, ataxia, and areflexia.^2^, ^6^ The axonal subtype or acute motor axonal neuropathy (AMAN) makes up roughly 5% of GBS cases and presents in a similar pattern as AIDP albeit with a faster progression and greater severity of motor weakness, ataxia, and areflexia.^2^, ^8^ The severity of the AMAN subtype may have to do with its associated antiganglioside antibodies (specifically the anti‐Ga1NAc‐GD1a/anti‐GD1a) which target periaxonal membranes of motor nerves at the nodes and paranodes.^2^ Although our patient did not present with the typical ascending pattern of motor weakness associated with the AMAN subtype, he did experience a rapid spread of weakness to all four limbs which evolved into complete flaccid paralysis, total areflexia in the extremities, absent bulbar or ocular involvement, and positive CSF findings for the anti‐GD1a antibodies, which led us to believe he was most likely suffering from an atypical form of classical AMAN GBS.

## 5 MILLER‐FISHER SYNDROME

By definition, the variant form Miller‐Fisher syndrome (MFS) of GBS does not include limb weakness (a characteristic that clinically separates this variant from pharyngeal‐cervical‐brachial syndrome). Instead, patients present with a triad of ophthalmoplegia, ataxia, and areflexia.^1^, ^2^, ^6^ As our patient did have severe limb involvement and did not have ophthalmoplegia, MFS (or a MFS/classical GBS crossover) was not highly suspected in our patient. Furthermore, our patient did not test positive for the antiganglioside antibody most often associated with the MFS variant, namely the anti‐GQ1b antibody^1^, ^2^—found positive in 80%‐85% of MFS patients.^1^

## 6 PHARYNGEAL‐CERVICAL‐BRACHIAL SYNDROME

Pharyngeal‐cervical‐brachial syndrome (PCB) typically presents as a rapid progression from ptosis, to weakness in the facial, pharyngeal, neck, and arm muscles with associated areflexia.^1^, ^2^, ^6^ Although our patient never developed bulbar palsy during his hospital course (rather, weakness in his diaphragm that required intubation and ventilation), the fact that he had initial upper extremity weakness raised the question as to whether he was suffering from PCB syndrome or perhaps with a classical GBS crossover. Our patient tested negative for anti‐GT1a IgG antibodies,^1^ which are the antiganglioside associated with the PCB variant, making an atypical presentation of AMAN more likely, although at this point it is impossible to say with certainty whether there were any PCB variant involvement.

## 7 OTHER DIFFERENTIALS

In addition to GBS being a condition that relies heavily on clinical signs and symptoms for early diagnosis, GBS is also a diagnosis of exclusion—as there are other urgent etiologies of rapidly progressive weakness that require immediate attention, first and foremost being an acute stroke. Botulism toxicity is another condition that can mimic GBS and its variants (notably MFS and PCS).^9^ It is important to differentiate GBS from botulism, as the treatment for the two diseases is very different, and delays in treatment of either can have potential long‐term sequela, especially with regard to diaphragmatic weakness. Finally, multifocal motor neuropathy (MMN) is another asymmetric presenting autoimmune disease, in which sensation is spared (as in our patient), and GM1 IgM antibody titers are elevated; however, the course of MMN is often slowly progressive (unlike our patient), and patient will often require IVIG for years.^10^

## 8 TREATMENT

Once the diagnosis of GBS has been made on the grounds of clinical evidence, with the exclusion of other causes, treatment should begin. Due to our current understanding of GBS being driven by antibodies, immune therapy has been the gold standard of treatment since the 1970s.^5^ Plasma exchange (PLEX) introduced in 1978 and intravenous immunoglobulin (IVIG) in 1988 have shown through systematic reviews to be equivalent treatments for GBS when it comes to effectiveness, long‐term prognosis, and mortality improvement.^5^ The choice between PLEX and IVIG is usually dependant on the institution; however, because PLEX is typically less available and less convenient for patients in comparison with IVIG (central venous catheter vs a peripheral intravenous line, respectively), treatment with IVIG is typically more common.

As in our patient, between 9% and 30% of patients with GBS will develop respiratory compromise^11^ and in some cases may proceed to respiratory distress and/or failure due to diaphragmatic weakness. It is important therefore to monitor patients with any suspicion of GBS or its variants for signs of respiratory compromise, such as an increase in respiratory rate, decreased forced vital capacity (FVC), or decreased vital capacity (VC).^9^ Unfortunately, bedside FVC/VC measurement is not feasible in the majority of hospitals, and other more practical methods such as peak flow measurements and pulse oximetry have been shown to be inadequate means of monitoring.

Sharshar et al were able to determine six positive predictive signs for likely endotracheal intubation from 722 French patients with GBS from their study in 2003. The predictors included time from onset to admission <7 days (OR: 2.51), inability to cough (OR: 9.09), inability to stand (OR: 2.53), inability to lift the elbows or head (OR: 2.99 and 4.34, respectively), and an increase in liver enzymes (OR: 2.09).^10^ Sharshar et al advocated that patients with any one of these signs should be admitted to the intensive care unit for monitoring and observed that >85% of these patients admitted to the ICU with ≥4 of these signs eventually needed intubation.^12^

## 9 CONCLUSION

Guillain‐Barré syndrome as we currently understand is an antibody‐driven autoimmune disorder that targets axonal gangliosides. Classically, GBS presents with progressive motor weakness in the lower extremities with symmetric rostral progression, although different patterns and variants of weakness are known to exist. This report showcases a patient with rapidly progressive and severe motor weakness which began in the right upper extremity and descended asymmetrically to include all four limbs. Once other etiologies have been ruled out, it is important that clinicians consider the possibility of GBS in any patient with a pattern of rapidly progressive weakness, as a more definitive means of diagnosis such as electrophysiological studies may not be available in every hospital, and biological testing may take a lengthy time to return. It is therefore important to continue to report on atypical cases of GBS in order to broaden clinical knowledge, with the hope of timely initiation and prevention of long‐term ataxia and chronic motor deficits in GBS patients.

## CONFLICT OF INTEREST

None declared.

## AUTHOR CONTRIBUTION

JMG is principal author, AG is supporting author, AL is principal investigator.
